# Food Gels: New Trends, Applications, and Challenges in the Food Industry

**DOI:** 10.3390/gels11110899

**Published:** 2025-11-10

**Authors:** Georgiana Gabriela Codină, Adriana Dabija

**Affiliations:** Faculty of Food Engineering, Stefan cel Mare University, 720229 Suceava, Romania; adriana.dabija@fia.usv.ro

## 1. Introduction

Different types of gels may be used in food industry to improve the quality of food products from a technological, nutritional, and sensory point of view. These can be obtained using natural or synthetic gelling agents, such as gelatin, agar-agar, pectin, carrageenan, gums (xanthan, guar, gellan), and different types of modified starches [1,2]. Each type of gel has specific physicochemical characteristics, which benefit their different food purposes. The ability of these gelling agents to form three-dimensional networks enables the formation of gel matrices capable of retaining water within the food structure, reducing moisture loss during processing, storage, or thermal heating [3]. Additionally, gelling agents conduct an increase in the viscosity of the food products, leading to an improvement of their texture and sensory characteristics [4]. An important advantage of gels is their ability to prevent phase separation, a common issue in emulsified or dispersed food systems such as sauces, creams, and dressings, e.g., [5]. By stabilizing aqueous and fatty phases, gels help maintain product homogeneity during storage, thus preventing quality defects such as sedimentation, syneresis, or coalescence. These functional properties are particularly important in the formulation of products such as yogurts, sauces, gelled desserts, processed meats, and bakery products, where consistency, homogeneity, and shelf-life period are key factors for consumers’ acceptability and the product’s quality preservation throughout their supply chain [6]. Usually used for their rheological properties, such as for thickening, gelling, and stabilizing, gelling agents are now used more and more for the development of healthier and functional food formulations with specific purposes [7]. Gels based on natural hydrocolloids contribute to the fiber intake of the human body and to the controlled release of bioactive compounds. Pectin gels improve soluble fiber content from food products and can reduce the rapid absorption of sugars, with beneficial effects on carbohydrate metabolism in the human body [8]. Alginates obtained from algae can be used to encapsulate vitamins and essential fatty acids, protecting them from oxidative degradation [9]. Protein gels, such as those obtained from whey, gelatin, or vegetable proteins, contribute to an increase in protein content and may also maintain the textures of food products [10]. Starch-based gels, including resistant starches support digestive health and enhance satiety [11]. Also, gelling agents obtained from different mixes form proteins and polysaccharides may be used as hybrid gels with mechanical stability and the ability to encapsulate and release nutrients in a controlled manner [12]. Recent trends in the use of different gels in the food industry indicate the development of gelling agents such as hydrogels, oleogels, emulsion gels, or bigels for healthier food products [1,2,13]. Hydrogels are often used in deliberate fat reduction and nutrient control due to their ability to encapsulate bioactive compounds [14]. Oleogels are currently used in fat replacers, offering a healthier alternative by replacing saturated fats in food products while maintaining their sensory characteristics [15]. Emulsion gels are especially used in the development of reduced-fat food products, enhancing both stability and texture due their combination of emulsion and gel properties, stabilizing oil droplets within a gel matrix [16]. Bigels integrate emulsion and gel networks in a single gel and have the potential to encapsulate different bioactive compounds in their matrix, which may lead to the development of different functional foods [17]. Recent uses of gelling agents based on plants such as seaweed and legumes are a response to consumers’ demand for various plant ingredients that improve the nutritional value of food products [10]. New trends indicate the possibility of using different gels in the 3D printing of good quality food, from sensory and nutritional points of view [18]. The future development of different gel types will lead to the creation of healthier and functional food products. However, the high cost of gelling agents such as hybrid gels or oleogels may limit commercial applications. Furthermore, the increasing demand for clean-label ingredients can represent real challenges for obtaining the desired gelling agents. Also, the stability of gels and encapsulated bioactive compounds may limit their functional use. Addressing these challenges may be the key to developing gel types that enable us to obtain healthier and functional food products.

This Special Issue, “Recent Advances on the Use of Different Gel Types in the Food Industry,” provides a comprehensive overview of the current progress and emerging applications of diverse gel types in food science and technology. It highlights how various gel formulations contribute to improving food quality through technological processes and sensory and nutritional characteristics.

## 2. An Overview of Published Articles

This Special Issue, “Recent Advances on the Use of Different Gel Types in the Food Industry,” brings together eight research articles highlighting recent advancements in gel-based systems for food applications. These researchers explored new possibilities of food quality improvement, new gel types, and their possible uses in food industry, as well as different technological changes that may occur in food processes due to the behavior of different gels.

The paper “Temperature-mediated gel texture transformation in starch noodles: In respect of glass transition temperature T_g_′” by Hongxiao Liu et al. analyzes the impact of freezing temperature on the texture formation, mechanical properties, and microstructure of potato starch noodles (PSNs), high-concentration starch gels formed through gelatinization and retrogradation. Freezing below the glass transition temperature (Tg′) demonstrates significant changes in the mechanical and microstructural characteristics of PSNs, which leads to more disrupted structures, while temperatures near the Tg′ enhance hardness, cohesiveness, and springiness due to reduced ice crystal formation and increased starch chain mobility.

The paper “Polydextrose addition improves the chewiness and extended shelf-life of Chinese steamed bread through the formation of a sticky, elastic network structure” by Chang Liu et al. reports the effects of 3–10% polydextrose addition on steamed bread. Polydextrose addition significantly modified the dough structure and its rheological characteristics and improved bread quality, especially in terms of its dietary fiber content, chewiness, and shelf life.

The paper “Comparison of ultrasound- and microwave-assisted extraction techniques on chemical, technological, rheological, and microstructural properties of starch from mango kernel” by Luis Mieles-Gómez et al. investigates different technologies (conventional extraction, ultrasound-assisted extraction, and microwave-assisted extraction) for starch extraction in terms of the technological, chemical, pasting, gelling, and microstructural properties of starch from mango kernel. Ultrasound and microwave-assisted extraction led to a high extraction yield and new starch characteristics, which may increase its application in the food industry.

The paper “Self-reactive carbon dioxide absorbent with sodium carbonate-based hydrogel” by Jae Young Kim and Youn Suk Lee presents a new hydrogel with CO_2_ absorption functionality developed through the addition of polyacrylic acid sodium salt (PAAS) to a sodium carbonate solution. The use of PAAS improves conventional sodium carbonate-based CO_2_ absorbents, which require water for activation. Since the hydrogel contains water, it can react with CO_2_, and the inclusion of PAAS will improve CO_2_ absorption capacity.

The paper “Development and quality enhancement of fried fish cake prototype with transglutaminase, trehalose, and herbal oil for room temperature distribution” by Ye Youl Kim et al. presents a new prototype of fried fish cake with good quality and an extended shelf-life. Response surface regression analysis established optimal processing conditions for the prototypes, using whiteness, DPPH radical scavenging activity, and gel strength as dependent variables. Incorporating trehalose, transglutaminase, and herbal oils and using optimum processing conditions allow surimi-based products to be stored at room temperature, increasing their profitability.

The paper “Incorporation of locust bean gum and solid lipid microparticles as strategies to improve the properties and stability of calcium-rich soy protein isolate gels” by Thais C. Brito-Oliveira et al. investigates the addition of different levels of locust bean gum and solid lipid microparticles (SLMs) on the properties of calcium-rich soy protein isolate gel, such as in terms of its visual aspect, water-holding capacity (WHC), rheological properties, and microstructural organization. According to the data obtained, these additions indicate the possibility of improving the quality of calcium-rich soy protein isolate gels in order to be used as a new product prototype in food industry.

The paper “Characterization of beeswax and rice bran wax oleogels based on different types of vegetable oils and their impact on wheat flour dough technological behavior during bun making” by Sorina Ropciuc et al. analyzes different types of oleogels obtained from beeswax and rice bran wax and five types of oils. The study concluded that all oleogel types showed good quality characteristics for their use in the food industry and may improve the quality of bakery products since the rheological data indicate good mixing, pasting, and fermentation behavior of dough samples with the addition of oleogels in their recipe.

The paper “Preparation and characterization of boza enriched with nonfat dry milk and its impact on the fermentation process” by Ezgi Pulatsu et al. studies the impact of adding nonfat dry milk (NFDM) on boza technological process and quality. According to the physicochemical data obtained, the sample with 10% NFDM was reported as the best sample.

## 3. Conclusions

The research presented in this Special Issue highlights the possibility of developing new food products, such as boza-based ones, with the addition of different amounts of nonfat dry milk addition, bakery products using different oleogels types or polydextrose, or fish products combining sugars, enzymes, and herbal oils. It investigates the possibility of obtaining new gel systems in food industry that may be improved in terms of their technological process or recipe. Temperature was shown to have a significant impact on gel structure and the texture of starch-based products, while some extraction techniques, such as ultrasound, improved starch quality and processing efficiency. Meanwhile, sodium carbonate-based hydrogels emerged as promising materials for sustainable CO_2_ absorption. These studies reflect the new trends in developing technologically optimized gel-based systems to obtain healthier food products with good quality characteristics.
